# SPAG17 mediates nuclear translocation of protamines during spermiogenesis

**DOI:** 10.3389/fcell.2023.1125096

**Published:** 2023-09-12

**Authors:** Clara Agudo-Rios, Amber Rogers, Isaiah King, Virali Bhagat, Le My Tu Nguyen, Carlos Córdova-Fletes, Diego Krapf, Jerome F. Strauss, Lena Arévalo, Gina Esther Merges, Hubert Schorle, Eduardo R. S. Roldan, Maria Eugenia Teves

**Affiliations:** ^1^ Department of Obstetrics and Gynecology, Virginia Commonwealth University, Richmond, VA, United States; ^2^ Department of Biodiversity and Evolutionary Biology, Museo Nacional de Ciencias Naturales, CSIC, Madrid, Spain; ^3^ Departamento de Bioquímica y Medicina Molecular, Facultad de Medicina, Universidad Autónoma de Nuevo León, Monterrey, Mexico; ^4^ Department of Electrical and Computer Engineering, Colorado State University, Fort Collins, CO, United States; ^5^ Department of Obstetrics and Gynecology, Perelman School of Medicine, University of Pennsylvania, Philadelphia, PA, United States; ^6^ Department of Developmental Pathology, Institute of Pathology, University Hospital Bonn, Bonn, Germany

**Keywords:** protamine, SPAG17, spermatogenesis, nucleocytoplasmic transport, spermiogenensis

## Abstract

Protamines (PRM1 and PRM2) are small, arginine-rich, nuclear proteins that replace histones in the final stages of spermiogenesis, ensuring chromatin compaction and nuclear remodeling. Defects in protamination lead to increased DNA fragmentation and reduced male fertility. Since efficient sperm production requires the translocation of protamines from the cytoplasm to the nucleus, we investigated whether SPAG17, a protein crucial for intracellular protein trafficking during spermiogenesis, participates in protamine transport. Initially, we assessed the protein-protein interaction between SPAG17 and protamines using proximity ligation assays, revealing a significant interaction originating in the cytoplasm and persisting within the nucleus. Subsequently, immunoprecipitation and mass spectrometry (IP/MS) assays validated this initial observation. Sperm and spermatids from *Spag17* knockout mice exhibited abnormal protamination, as revealed by chromomycin A3 staining, suggesting defects in protamine content. However, no differences were observed in the expression of *Prm1* and *Prm2* mRNA or in protein levels between testes of wild-type and *Spag17* knockout mice. Conversely, immunofluorescence studies conducted on isolated mouse spermatids unveiled reduced nuclear/cytoplasm ratios of protamines in *Spag17* knockout spermatids compared to wild-type controls, implying transport defects of protamines into the spermatid nucleus. In alignment with these findings, *in vitro* experiments involving somatic cells, including mouse embryonic fibroblasts, exhibited compromised nuclear translocation of PRM1 and PRM2 in the absence of SPAG17. Collectively, our results present compelling evidence that SPAG17 facilitates the transport of protamines from the cytoplasm to the nucleus.

## 1 Introduction

Spermiogenesis, the last phase in the process of spermatogenesis, is a vital process for male germ cell differentiation, and encompasses a series of events crucial for sperm maturation and fertility. It involves various intricate steps such as sperm head elongation, nuclear remodeling, chromatin condensation, and flagellar development (Teves and Roldan, 2022). Notably, during the final stages of spermiogenesis, nuclear histones undergo replacement by two types of protamines, namely, PRM1 and PRM2. These protamines are proteins rich in arginine and cysteine residues, contributing to the structural and functional transformation of sperm chromatin (Teves and Roldan, 2022; Balhorn, 2007).

Protamines stand as the most prevalent nuclear proteins within sperm. These proteins, exclusive to male germ cells, play a pivotal role in packaging the paternal genome, gradually replacing histones during spermiogenesis (Oliva, 2006; Balhorn et al., 2018; Arévalo et al., 2022a). Numerous studies have shown that protamine-related changes can directly impact sperm DNA and the nucleus. Aberrations in DNA compaction by protamines can cause DNA fragmentation, alterations in seminal parameters, ultimately leading to reduced male fertility and the onset of genetic mutations in offsprings (Aitken et al., 2009; Andraszek et al., 2014; Ni et al., 2016). The significance of chromatin compaction facilitated by protamines has been highlighted by several studies on protamine deficient mice (Arévalo et al., 2022b; Moritz and Hammoud, 2022). Loss of both PRM1 alleles leads to infertility whereas loss of one PRM1 allele results in severe reduction of sperm motility and male subfertility (Merges et al., 2022). Sperm from *Prm1* ^+/−^ and *Prm1* ^−/−^ mice contained high levels of incompletely processed PRM2 which suggests that PRM1 is needed for PRM2 processing (Merges et al., 2022). Conversely, while *Prm2* ^*−/−*^ mice are infertile, the heterozygous loss of PRM2 does not lead to subfertility (Schneider et al., 2016). Unprocessed PRM2 seems to play a distinct role related to the elimination of intermediate DNA-bound proteins and the incorporation of both protamines into chromatin (Arévalo et al., 2022a). Hence, although both protamines are crucial for the production of functional sperm, they appear to perform distinct functions beyond simple DNA compaction (Arévalo et al., 2022b).

PRM1 is present in all mammals and is synthesized directly as a mature protein. In contrast, PRM2 is primarily found in rodents and primates and is synthesized as a precursor that is processed by sequential cleavage to its mature form (Balhorn et al., 2018; Arévalo et al., 2022b). These male germ cell specific proteins are responsible for DNA hyper-condensation and chromatin structural reorganization thus protecting DNA strands from possible breaks and preserving the integrity of the genome (Silva et al., 2021). This implies that any protamine-related changes can directly impact sperm DNA and nucleus, thus affecting sperm function (Andraszek et al., 2014).

Protamines are expressed in haploid male germ cells (Teves and Roldan, 2022). During spermiogenesis, protamine genes are expressed soon after completion of meiosis, in round spermatids (Hecht et al., 1986a; b). Subsequently, transcripts are stored as cytoplasmic ribonucleoprotein particles for several days until protein synthesis, which takes place in elongating spermatids (Kleene et al., 1984; Kleene, 1989). Most studies on protamines have focused on their interaction with nuclear chromatin and the relevance of chromatin reorganization and nuclear reshaping for sperm morphology and performance. However, very little is known regarding mechanisms of protamine transport from the cytoplasm to the nucleus.

Protein transport, including the trafficking of essential proteins such as protamines, plays a crucial role in spermiogenesis. During steps 8–14 of mouse spermiogenesis, a transient structure called the manchette, facilitates this transport process (Teves et al., 2020). The manchette comprises microtubules and actin filaments that act as tracks for intracellular protein trafficking through multi-subunit complexes, a mechanism known as intramanchette transport (IMT) (Kierszenbaum, 2002). Despite the significance of the manchette in protein trafficking, our understanding of its proteomics and its association with nucleocytoplasmic protein transport remains limited (Pleuger et al., 2020; Teves et al., 2020).

In a recent study by Kazarian et al. (2018), the Sperm-associated antigen 17 (SPAG17) was found to be expressed in testicular germ cells during the late stages of sperm development and was shown to localize to the manchette, contributing to protein trafficking. Notably, proteins known to be associated with the manchette and transported through it failed to properly localize to the manchette in *Spag17* knockout elongating spermatids, remaining in the cytoplasm instead. Additionally, electron microscopy evaluation of testicular preparations revealed multiple phenotypes, including defects in chromatin compaction and nuclear remodeling. These findings suggest a potential association between SPAG17 and the transport or dysfunction of protamines.

To further investigate the role of SPAG17 in protein transport during spermiogenesis, in the present study, we aimed to determine the interaction between SPAG17 and protamines, as well as its influence on the nuclear translocation of PRM1 and PRM2. By examining these protein interactions, we seek to gain insights into the role of SPAG17 in facilitating protamine transport and its impact on the process of spermiogenesis. This will contribute to a better understanding of the mechanisms underlying protein trafficking during spermiogenesis and shed light on the potential implications for male fertility.

## 2 Materials and methods

### 2.1 Animals

All animal studies were conducted in accordance with protocol AM10297 approved by the Virginia Commonwealth University Institutional Animal Care and Use Committee. Heterozygous *B6N(Cg)-Spag17 tm1b*(*KOMP*)*Wts1/J* (Stock No. 026485) mice from Jackson Laboratories were used to generate homozygous (*Spag17/Sox2-Cre*) mice with disrupted expression of the *Spag17* gene. The disruption in the gene was accomplished by the precise insertion of the L1L2_Bact_P cassette on Chromosome 3. This cassette comprised an array of genetic elements, including FRT sites, a lacZ sequence, and loxP sites. These loxP sites strategically flanked the critical exon, allowing for precise control and manipulation of gene expression. To generate the strain, the construct was introduced into JM8. N4 embryonic stem (ES) cells derived from the C57BL/6N lineage. After confirming the correct targeting of the ES cells, they were injected into blastocysts obtained from *B6(Cg)-Tyrc-2J/J* mice (Stock No. 58). The resulting chimeric males were then bred to C57BL/6NJ females (Stock No. 005304), followed by mating with *B6N.Cg-Tg(Sox2-cre)1Amc/J* mice (Stock No. 014094) to excise the floxed neomycin cassette and critical exon sequences. This breeding strategy ensured the removal of unwanted transgenic elements and further refined the genetic background of the offspring. After a deletion in exon 5 a premature stop codon leads to absent SPAG17 expression. Male fertility phenotype and experiments validating the deletion of this gene were reported previously in Kazarian et al. (2018). The wild-type mice used as controls share the same genetic background as the *Spag17/Sox2-Cre* knockout (KO) mice. To ensure consistency and minimize genetic variations, we typically use mice from the same litter or mice derived from the same breeding line. The total number of animals used for these studies are wild-type n = 20 and knockout n = 22.

### 2.2 Mixed germ cells isolation

Testes from adult wild-type (n = 13) and *Spag17/Sox2-Cre* (n = 14) mouse line were de-capsulated and placed into isolation buffer containing 5 mL DMEM (Gibco, Life Technologies Corporation, Grand Island, NY, United States), 1 μg/mL DNase I (Sigma-Aldrich, St. Louis, MO, United States) and 0.5 mg/mL collagenase IV (Sigma-Aldrich, St. Louis, MO, United States), and then incubated for 30 min at 32°C to dissociate the tissue. The digested tissue was filtered through a 40 μm cell strainer to remove somatic cells. Then, the separated suspension containing mixed germ cells (from spermatogonia to elongated spermatids) was centrifuged for 5 min at 1,000 rpm and 4°C and washed twice with 5 mL PBS.

### 2.3 Proximity ligation assay (PLA)

Mixed germ cells isolated from adult wild-type (n = 3) and *Spag17/Sox2-Cre* knockout (n = 3) mice were fixed in 4% paraformaldehyde/PBS (containing 0.1 M sucrose) for 15 min at room temperature. After 3 washes with PBS, cells were resuspended in PBS, spread on SuperFrost/Plus slides (Fisher Scientific, Pittsburgh, PA, United States), and used for protein-protein interaction determination by Duolink^®^ PLA (Sigma-Aldrich, St. Louis, MO, United States) following the manufacturer’s instructions (Supplementary Figure S1). Anti-PRM1, anti-PRM2 and anti-SPAG17 were used as primary antibodies. Anti-α and anti-β tubulin antibodies were used as positive controls (Supplementary Table S1). Three independent PLAs experiments were performed for each protein-protein interaction.

### 2.4 Immunoprecipitation and mass spectrometry

Germ cells from adult wild-type (n = 3) and *Spag17/Sox2-Cre* knockout (n = 3) mice were isolated as described above and treated with NP40 lysis buffer (50 mM TRIS pH 7.4, 250 mM NaCl, 5 mM EDTA, 50 mM NaF, 1 mM Na_3_VO_4_, 1% NP40, 0.02% NaN_3_ and protease inhibitor cocktail) for 30 min at 4°C. The cell lysate was then sonicated 10 times in 30 s intervals on ice and cleared by centrifugation. Protein concentration was determined using the Lowry assay protocol.

For each sample, a volume of 100 μL of Dynabeads M-280 sheep anti-rabbit IgG (Invitrogen) was pre-incubated with and without the previously validated anti-SPAG17 antibody (Zhang et al., 2005; Teves et al., 2015; Kazarian et al., 2018) at a dilution of 1/100 and rotated overnight at 4°C. The beads were subsequently washed seven times with DPBS (Gibco, Life Technologies Corporation, Grand Island, NY, United States). For immunoprecipitation (IP), 200 μg of total protein from each cell lysate was incubated with the pre-treated Dynabeads and left to incubate overnight at 4°C. The Dynabeads were then washed 14 times with DPBS at 4°C. The proteins were boiled in 4x Laemmli buffer for 10 min to separate them from the Dynabeads. The supernatant from each sample was loaded onto a respective 10% acrylamide gel well and electrophoretically separated at 80 V. As a control for peptides visualization gels were stained with Coomassie blue for 2 h (Supplementary Figure S2). Next, each well line was cut and submitted to liquid chromatography with tandem mass spectrometry (LC-MS/MS) using the Thermo Electron Q Exactive HF mass spectrometer system.

The gel pieces were transferred to a siliconized tube and washed in 200 µL 50% methanol. They were dehydrated in acetonitrile, rehydrated in 30 µL of 10 mM dithiolthreitol (DTT) in 0.1 M ammonium bicarbonate and reduced at room temperature for 0.5 h. The DTT solution was removed, and the sample alkylated in 30 µL 50 mM iodoacetamide in 0.1 M ammonium bicarbonate at room temperature for 0.5 h. The reagent was removed and the gel pieces dehydrated in 100 µL acetonitrile. The acetonitrile was removed and the gel pieces rehydrated in 100 µL 0.1 M ammonium bicarbonate and the pieces were let to dried by vacuum centrifugation. Rehydration was performed in 20 ng/μL trypsin in 50 mM ammonium bicarbonate on ice for 30 min. Any excess enzyme solution was removed, and 20 µL 50 mM ammonium bicarbonate was added. Next, samples were digested overnight at 37°C and the peptides formed were extracted from the polyacrylamide in a 100 µL aliquot of 50% acetonitrile/5% formic acid. The extract was evaporated to 100 µL for MS analysis. The LC-MS system consisted of a Thermo Electron Q Exactive HF mass spectrometer system with an Easy Spray ion source connected to a Thermo 75 μm × 15 cm C18 Easy Spray column (through pre-column). 0.8 µg equivalent of the original extract was injected and the peptides eluted from the column by an acetonitrile/0.1 M acetic acid gradient at a flow rate of 0.3 μL/min over 2 h. The nanospray ion source was operated at 1.9 kV. The digest was analyzed using the rapid switching capability of the instrument acquiring a full scan mass spectrum to determine peptide molecular weights followed by product ion spectra (Top10 HCD) to determine amino acid sequence in sequential scans. This mode of analysis produces approximately 30,000 MS/MS spectra of ions ranging in abundance over several orders of magnitude.

Data were analyzed by database searching using the Sequest search algorithm against Uniprot Mouse. For validation of MS/MS-based peptide and protein identifications, Scaffold 5 (Proteome Software Inc., Portland, OR) was used. Peptide identification was accepted if it could be established at >95% probability by the Peptide Prophet algorithm with Scaffold delta-mass correction.

### 2.5 Sperm isolation

Sperm from adult wild-type (n = 3) and *Spag17/Sox2-Cre* knockout (n = 4) mice were collected from the caudae epididymides. Briefly, spermatozoa were collected by making several cuts into the cauda epididymis with surgical scissors in 1 mL of pre-warmed (35°C) PBS or Medium 199 (Gibco, Life Technologies Corporation, Grand Island, NY, United States) supplemented with 4 mg/mL BSA (Sigma, St. Louis, MO) and allowing the sperm to swim out from the tissue for 10 min. In the case of *Spag17/Sox2-Cre* knockout samples, additional gentle pipetting up and down of the suspension was performed to improve collection of sperm since lack of progressive movement is a phenotype present in these mice (Kazarian et al., 2018). As a control, aliquots of sperm suspension were used to assess % of sperm with progressive movement (WT = 70 ± 7.0%; KO = 0 ± 0.0%, Supplementary Movie S1, S2) and viability after trypan blue staining (Serafini et al., 2014) (No. T8154, Sigma, St. Louis, MO, WT = 87 ± 5.5%; KO = 70 ± 10.2%; Supplementary Figure S3) and observation using phase contrast or bright light microscopy, respectively.

### 2.6 Protamination assay

Assessments were performed in cells collected from adult wild-type (n = 3) and *Spag17/Sox2-Cre* knockout (n = 4) mice. The methodology used for the evaluation of protamine deficiency with chromomycin A3 (CMA3) was based on protocols used by Lolis et al. (1996) and Castro et al. (2018). Briefly, two smears, one for isolated sperm and one for isolated testicular germ cells, were prepared using 20 μL of sample and air-dried. Each slide was fixed in Carnoy’s solution (ethanol:acetic acid, 3:1) for 5 min at 4°C and air-dried. For each slide 100 μL of 0.25 mg/mL CMA3 (Thermo Fisher Scientific Inc., Waltham, MA, United States) in McIlvain’s buffer (7 mL citric acid 0.1M + 33 mL Na_2_HPO_4_ 0.2 M, pH 7.0, containing 10 mM MgCl_2_ and 1% DMSO to facilitate dissolution), was added and slides were incubated in the dark for 20 min at room temperature. Slides were rinsed in McIlvain’s buffer and mounted with a drop of buffered glycerol (glycerol:phosphate buffer 0.2 M, pH 7.4, 1:1) for microscopic analysis. Images were captured by Zeiss LSM700 confocal microscope. Quantification of CMA3 positive cells was performed using the combination of bright and green fluorescent field. A total of 100–200 sperm or spermatids per slide were counted. Cells with correct protamination (negative green fluorescence) and abnormal protamination (positive bright green fluorescence) were identified and quantified.

### 2.7 Immunofluorescence staining

Isolated mixed germ cells from adult wild-type (n = 4) and *Spag17/Sox2-Cre* knockout (n = 4) mice were fixed in 4% paraformaldehyde/PBS (containing 0.1 M sucrose) for 15 min at room temperature. After 3 washes with PBS, cells were resuspended in PBS and spread on SuperFrost/Plus slides (Fisher Scientific, Pittsburgh, PA, United States) and used for immunofluorescence staining. Staining was conducted to examine the patterns of protein localization. Slides were blocked with blocking solution containing 10% goat serum (Vector Laboratories, Inc., Burlingame, CA, United States), 3% BSA (Sigma-Aldrich, St. Louis, MO, United States) and 0.2% Triton X-100 (Fisher Scientific, New Jersey, NY), and incubated at room temperature for 1 h. The respective primary antibodies (Supplementary Table S1) were diluted in the same blocking solution and incubated overnight at 4°C. Following incubation, samples were washed 3 times (10 min each) with PBS and incubated with secondary antibody (Alexa Fluor 594-conjugated anti mouse IgG; Supplementary Table S1) at room temperature (in the dark) for 1–2 h. After 3 washes with PBS, slides were mounted with VectaMount with DAPI mounting media (Vector laboratories, Inc., Burlingame, CA, United States) and sealed with a coverslip and nail polish. Images were captured with a Zeiss LSM700 confocal laser-scanning microscope and analyzed using ImageJ.

### 2.8 Cell culture and transfection

Mouse embryonic fibroblasts (MEFs) were isolated from wild-type (n = 4) and *Spag17/CMV-Cre* knockout (n = 4) E12.5 embryos as previously reported (Teves et al., 2015). They were then cultured in DMEM medium (Gibco, Life Technologies Corporation, Grand Island, NY, United States) supplemented with 1 mg/ml L-glutamine (Gibco, Life Technologies Corporation, Grand Island, NY, United States) and 10% FBS (R&D Systems, Minneapolis, MN, United States). At about 50%–60% confluency, cells were transfected with 1 μg mouse pPrm1-mCherry-N1 or mouse pPrm2-EGFP-N3 expressing vectors (Arévalo et al., 2022a) (Supplementary Figure S4) using Continuum (Gemini Bio-Products, West Sacramento, CA) to express PRM1 and PRM2, respectively. 1 μg of pmCherry-N1 or pEGFP-N3 were used as control vectors. After 6 h, transfection medium was replaced by culture medium for two time points 24 and 48 h. Then cells were fixed in 10% formalin (Sigma, St. Louis, MO, United States), mounted with VectaMount with DAPI mounting media (Vector laboratories, Inc., Burlingame, CA, United States) and sealed with a coverslip and nail polish. Four independent experiments were performed. *Spag17/CMV-Cre* KO MEFs were challenging to transfect, which led to a smaller number of transfected cells in this genotype than in the wild-type. For quantification, we analyzed approximately 60–80 transfected wild-type MEFs and 25–30 transfected knockout MEFs per treatment and experiment. Images were captured with a Zeiss LSM700 confocal laser-scanning microscope and analyzed using ImageJ.

### 2.9 Gene expression of protamines

RNA extraction, cDNA synthesis, quantitative PCR and analysis of expression data was carried out as described previously (Lüke et al., 2014). Briefly, RNA was extracted from adult wild-type (n = 4) and *Spag17/Sox2-Cre* knockout (n = 5) mouse testes using TRIzol (Invitrogen, Carlsbad, CA, United States), following the manufacturer’s recommendations. RNA concentration and purity were determined using a NanoDrop 1,000 spectrophotometer (Thermo Scientific, Washington, DE, United States). Total RNA was reverse-transcribed with RETROscript kit (Ambion, Austin, TX, United States) according to the manufacturer’s instructions. Expression levels of *Prm1* and *Prm2* in wild-type and *Spag17* knockout mice was determined using a realplex mastercycler (Eppendorf, Hamburg, Germany). Primers used were those designed by Lüke et al. (2014) for mouse transcripts. qPCR reactions were run in 96-well plates with an end volume of 20 µL per sample containing 10 µL SiTaq universal SYBR green supermix (BIO-RAD, Hercules, CA, United States), 300 nM of each primer and 50 ng/mL of cDNA. The conditions of the thermocycler program consisted of an initial denaturation of 95°C for 2 min, 40 cycles of 95°C for 15 s and an annealing and elongation stage of 62°C for 1 min. Melt curve analysis was performed at the end of each run to check for multiple peaks, indicative of non-specific amplification. Cycle threshold data (CT) were normalized relative to 18SrRNA for each plate (∆CT). Data were transformed by adding a constant based on the lowest ∆CT value. Expression ratios and percentages were calculated from transformed individual ∆CT values and median values were obtained for each group.

### 2.10 PRM1 and PRM2 protein expression in testes

Basic proteins were extracted from testes as described in Soler-Ventura et al. (2018) with slight modifications (n = 3 per genotype). Briefly, frozen testes were decapsulated, homogenized and washed in PBS. The pellet was resuspended in buffer containing 1 M Tris pH 8, 0.5 M MgCl_2_ and 5 μL Triton X-100 and then treated with 1 mM PMSF in water inducing cell lysis. Subsequently the samples were treated with EDTA, DTT and GuHCl inducing DNA denaturation. The samples were then incubated at 37°C for 30 min in the presence of 1% vinylpyridine for mouse protamine separation on the subsequent protein gel. The amount of vinylpyridine used was slightly increased compared to the published protocol (0.8%), which improved the separation of the protamine bands. DNA was then precipitated by addition of ethanol and separated from the sample by centrifugation. Basic proteins were then dissolved in 0.5 M HCl and precipitated with TCA, followed by acetone washes and drying. The precipitated proteins were resuspended in sample buffer containing 5.5 M urea, 20% β-mercapto-ethanol and 5% acetic acid.

The samples were then run on a pre-electrophorized acid-urea polyacrylamide gel (AU-PAGE) (2.5 M urea, 0.9 M acetic acid, and 15% acrylamide/0.1% N,N′-Methylene bis-acrylamide, TEMED and APS). The extracted basic proteins migrate towards the negative pole at 110 V for 2 h and 10 min. The gels were stained with Coomassie Brilliant Blue (Sigma Aldrich, Taufkirchen, Germany) using standard procedures. The two main protamine bands can be observed in the bottom of the gel with mature-PRM2 corresponding to the upper and PRM1 the lower band (Ishibashi et al., 2010; Soler-Ventura et al., 2018; Arévalo et al., 2022). PRM2 precursor bands can be observed in the lower part of the gel above the mature-PRM2 band (Yu et al., 2000; Mateo et al., 2011; Arévalo et al., 2022). In the upper half of the gel, bands corresponding to other basic nuclear proteins, including histones can be found (see Soler-Ventura et al., 2018). The densities of Coomassie stained bands were analyzed using ImageJ (1.52k, Schneider et al., 2012). The protamine content was quantified relative to the whole lane for each individual to ensure comparability. The ratio between PRM1 and PRM2 was calculated using the respective band density in each lane.

### 2.11 Image analysis

In order to differentiate the stages of various spermatids within a heterogeneous population of germ cells, we examined the cellular topological morphology using microscopy to categorize each stage. This evaluation encompasses the analysis of nuclear positioning within the cytoplasm, the configuration of the acrosome, and the presence or absence of the manchette structure. The nuclear/cytoplasmic ratios for PRM1 and PRM2 were computed using ImageJ software to investigate protamine translocation across various spermiogenesis stages. Total cell area was established from bright field images. Immunofluorescence images with blue (nucleus) and red (PRMs) channels were separated. The blue channel determined the total nuclear area, while the red channel was used to quantify fluorescence intensity for PRMs. Fluorescence in the cytoplasmic region was derived by subtracting nuclear fluorescence from the fluorescence in the entire cell, then divided by the respective area to normalize. Subsequently, the nucleus’s fluorescence intensity was divided by cytoplasmic fluorescence intensity (N/C ratio). The percentage of protamines in MEF nuclei was calculated by assessing fluorescence intensity as described. After adjusting for area effects, nuclear values were multiplied by 100 and divided by total cell fluorescence.

### 2.12 Statistical analysis

Statistical analysis was performed using GraphPad Prism 8 software (GraphPad Software, San Diego, CA). The data are presented as means ± standard error of the mean (SEM). To compare the means between two groups, Student’s t-test was utilized. A significance level of *p* < 0.05 was considered to indicate statistically significant differences between the samples.

## 3 Results

### 3.1 Interaction of SPAG17 and protamines

**FIGURE 1:**
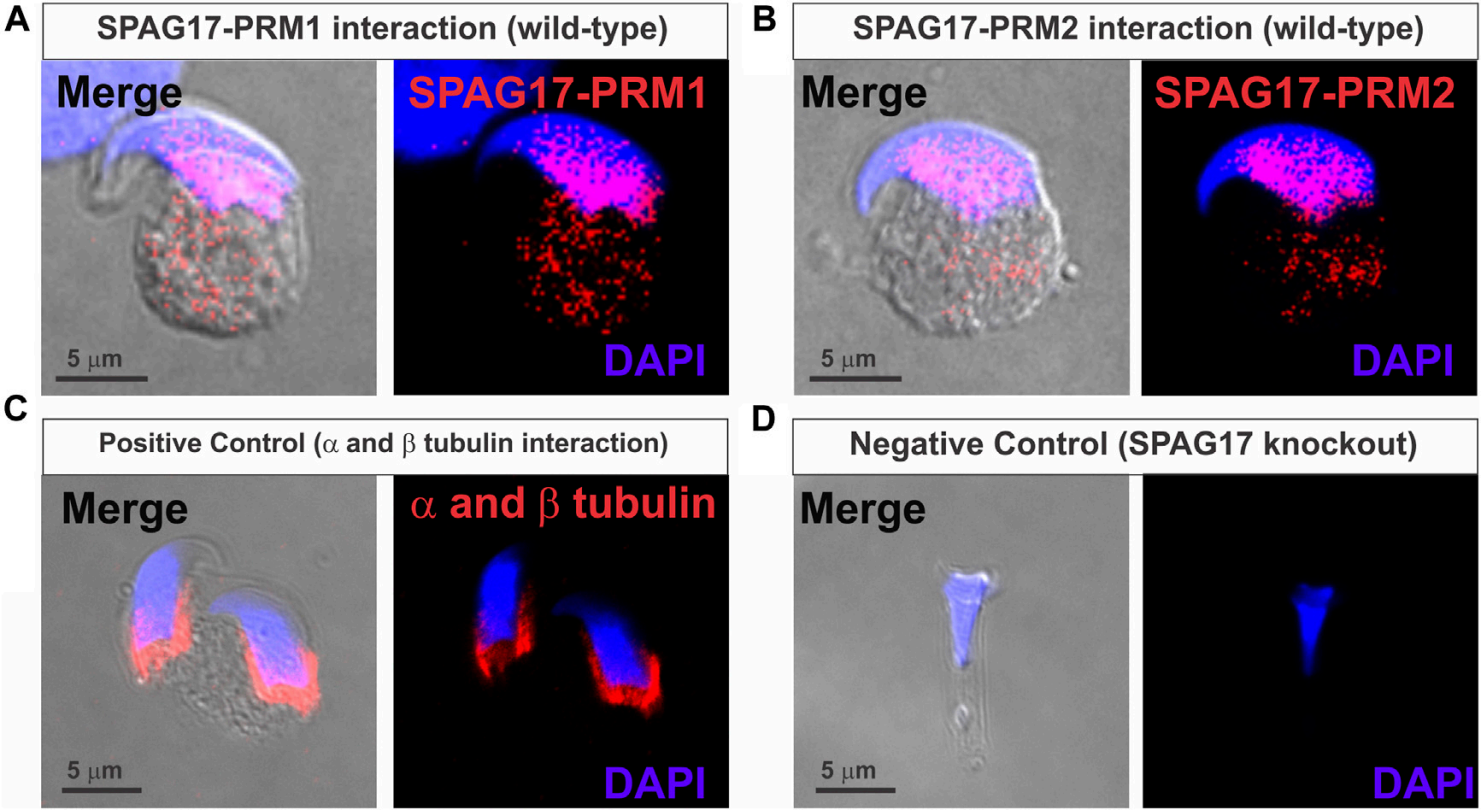
Proximity ligation assay (PLA) showing interaction of SPAG17 and protamines in mouse elongating spermatids. **(A)** Representative image showing interaction of SPAG17 and PRM1 in wild-type elongating spermatid step 12–13. **(B)** Representative images showing interaction of SPAG17 and PRM2 in wild-type elongating spermatid step 12–13. **(C)** Representative images showing positive control using anti-α and anti-β tubulin primary antibodies in wild-type elongating spermatids step 11. **(D)** Representative images showing lack of interaction in *Spag17* knockout elongating spermatid step 14, which lack SPAG17 protein, when anti-SPAG17 and anti-PRM2 antibodies were used. Images were collected from 3 independent PLAs experiments.

The SPAG17 protein has recently been shown to play a role in protein transport via the manchette. Importantly, *Spag17* knockout mice have defects in chromatin compaction (Kazarian et al., 2018), suggesting influence of SPAG17 in protamines content or function. Thus, we investigated SPAG17-protamine interactions. By using proximity ligation assay (PLA), the protein-protein interactions between SPAG17 and PRM1 and PRM2 were assessed in elongating spermatids. An intense fluorescent signal was observed using anti-SPAG17 and anti-PRM1 or PRM2 antibodies in wild-type spermatids. Interaction starts in the cytoplasm (Supplementary Figure S5A, B) and then moves to the nucleus following the nuclear translocation steps (Figures 1A,B). Positive controls using anti-α and β-tubulin antibodies also showed an intense fluorescent signal (Figure 1C), while anti-SPAG17 and anti-PRM1 or PRM2 antibodies did not show protein-protein interaction in *Spag17* knockout spermatids, as expected, due to lack of SPAG17 expression in these cells (negative control, Figure 1D; Supplementary Figure S5C).

Next, interaction of PRM1 and PRM2 with SPAG17 was confirmed by immunoprecipitation assay using anti-SPAG17 antibody and mass spectrometry. SPAG17, PRM1 and PRM2 were detected in germ cells collected from wild-type mice. In contrast, these proteins were not detected in samples from *Spag17* knockout mice. Table 1 shows the list of peptides found for the three proteins.

**TABLE 1:** List of peptides identified for SPAG17, PRM1 and PRM2. These peptides were discovered through immunoprecipitation using an anti-SPAG17 antibody, followed by proteomic analysis using LC-MS techniques. The results highlight the presence of multiple peptides associated with SPAG17, PRM1, and PRM2.

Proteins	Peptides
SPAG17	AVMPPLEQEASRVVTSQGTVIK
	SELSSLF
	VVTSQGTVIK
	ISSENYEPLQTHLAAVR
	TEEERGNAADLLK
	AVMPPLEQEASR
	VFTFESLKL
	QLTNIPAPILEGPK
	TQSYLMQIK
	SASQNEIEDLIK
PRM1	RRRRSYTIRCK
	RSYTIR
	RRSYTIR
PRM2	SPSEGPHQGPGQDHEREEQGQGQGLSPERVEDYGR
	EEQGQGQGLSPER
	EEQGQGQGLSPERVEDYGR

### 3.2 Sperm and spermatids from *Spag17* knockout mice display abnormal protamination

**FIGURE 2:**
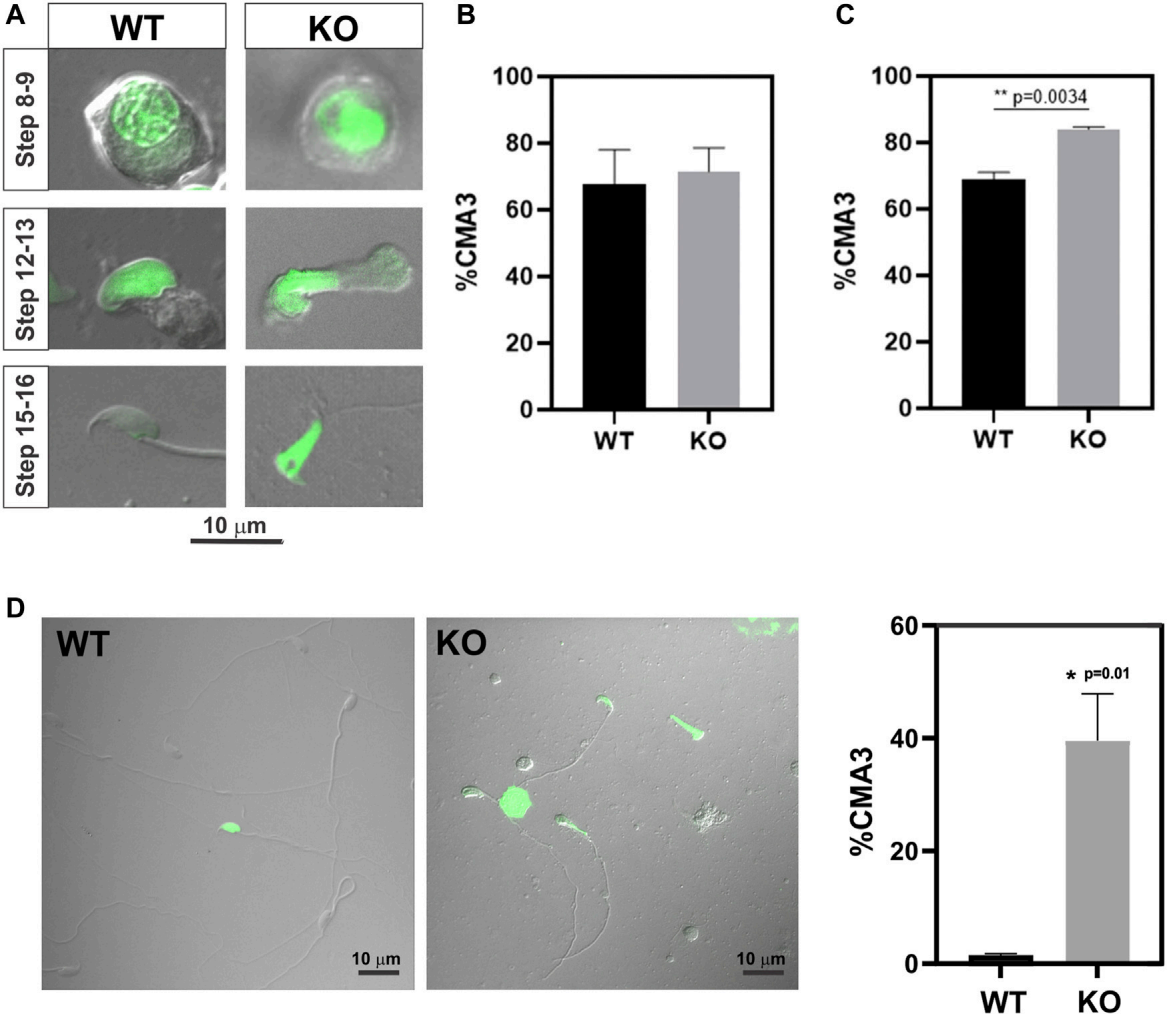
Abnormal protamination is observed in elongated spermatids and sperm from *Spag17* knockout mice. Spermatids and sperm were collected from the testes and cauda epididymis of wild-type (WT, n = 3) and *Spag17* knockout (KO, n = 4) mice, respectively, and stained with CMA3 to assess protamination. A total of 100–200 cells per sample were counted, and the percentage of CMA3-positive heads (green labeled) was calculated. **(A)** Representative images of spermatids at different steps. **(B)** Quantification of the percentage of CMA3-positive spermatids from step 8 to 16. **(C)** Quantification of the percentage of CMA3-positive spermatids from steps 15–16. **(D)** Representative images displaying CMA3-positive sperm and quantification of the percentage of CMA3-positive sperm. Results are means ± SEM. Significant differences were observed in comparison to WT, with * indicating *p* = 0.01 and ** indicating *p* = 0.0034.

Because previous evidence indicated reduced chromatin condensation in *Spag17* knockout spermatids, we investigated the levels of protamination in spermatids from steps 8 to 16. For this purpose, chromomycin A3, a fluorochrome that binds to guanine- and cytosine-rich sites and competes with protamines for binding to the minor groove of DNA (Pourmasumi et al., 2019), was used as an indicator of protamine-deficient chromatin decondensation (Lolis et al., 1996; Bizzaro et al., 1998; Castro et al., 2018; Ribas-Maynou et al., 2021) (Figure 2A). Due to the occurrence of protamination during the late stages of spermiogenesis, a substantial proportion of spermatids exhibited positive staining for CMA3 when considering spermatids from steps 8 to 16 collectively. Notably, there was no significant difference in CMA3-positive cells between wild-type and *Spag17* knockout spermatids (67.6% ± 10.5%, n = 3 and 71.5% ± 7.2%, n = 4 respectively) (Figure 2B). However, upon analyzing spermatids at steps 15 and 16, a significant difference (*p* = 0.0034) was observed between genotypes (Figure 2C). To further explore this protamination defect, we examined mature sperm. The analysis of cauda epididymal sperm revealed a significant difference in the percentage of CMA3-positive spermatozoa between the wild-type and *Spag17* knockout groups (WT: 1.5% ± 0.3%, n = 3; KO: 39.6% ± 8.3%, n = 4, *p* = 0.01), indicating a grater protamine deficiency in the absence of SPAG17 (Figure 2D).

**FIGURE 3:**
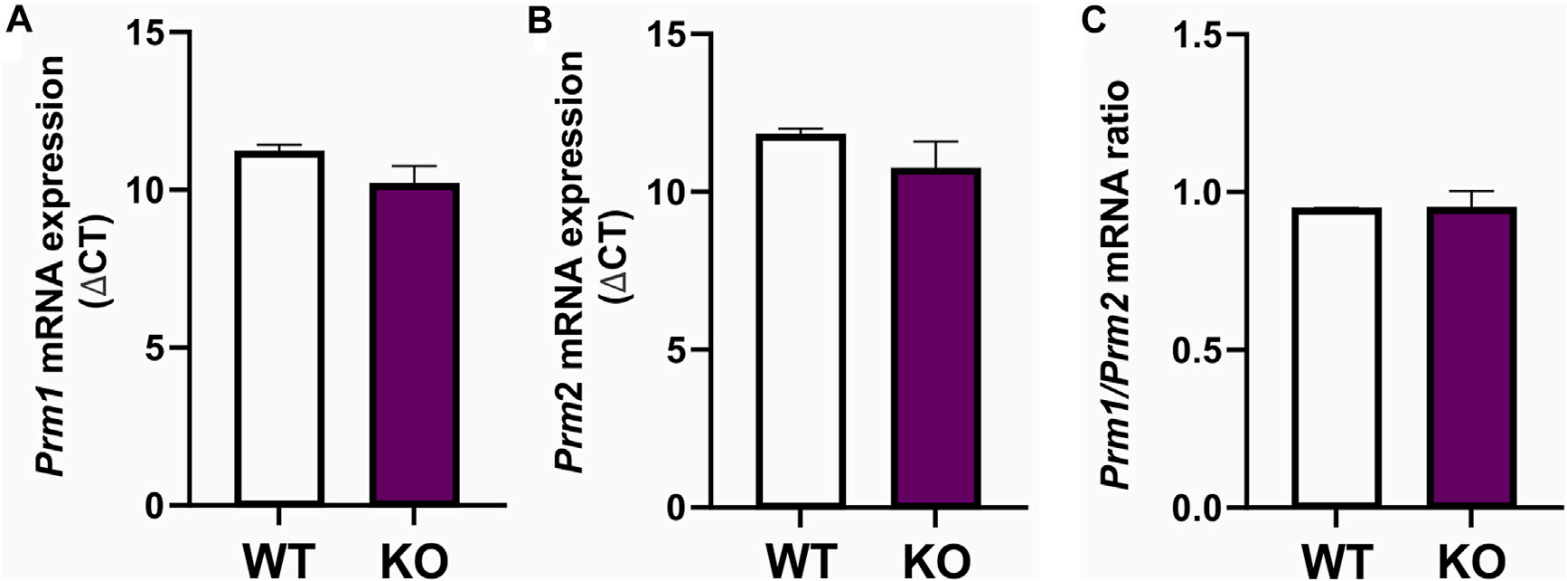
Expression of *Prm1* and *Prm2* mRNA is not different between testes from wild-type and *Spag17* knockout mice. Testes from adult wild-type (WT, n = 4) and *Spag17* knockout (KO, n = 5) mice were collected. Total RNA was extracted and used to determine *Prm1* and *Prm2* gene expression by qPCR using 18S rRNA as a housekeeping gene. **(A)** *Prm1* mRNA expression showed no differences between WT and KO testes; *p* = 0.17. **(B)** *Prm2* mRNA expression showed no differences between WT and KO testes; *p* = 0.31. **(C)** *Prm1/Prm2* mRNA ratio was not different between WT and KO testes. Results are means ± SEM, *p* = 0.96.

To determine if differences in protamination in *Spag17* knockout mice are related to gene expression levels, *Prm1* and *Prm2* mRNA expression was measured in wild-type and *Spag17* knockout testes by qPCR (Figure 3). No significant differences were found between the two groups for any of the protamines, nor for the ratio between them (*p* = 0.17 for *Prm1*, *p* = 0.31 for *Prm2* and *p* = 0.96 for *Prm1/Prm2* ratio), indicating that the loss of SPAG17 does not affect *Prm1* and *Prm2* expression at the mRNA level.

**FIGURE 4:**
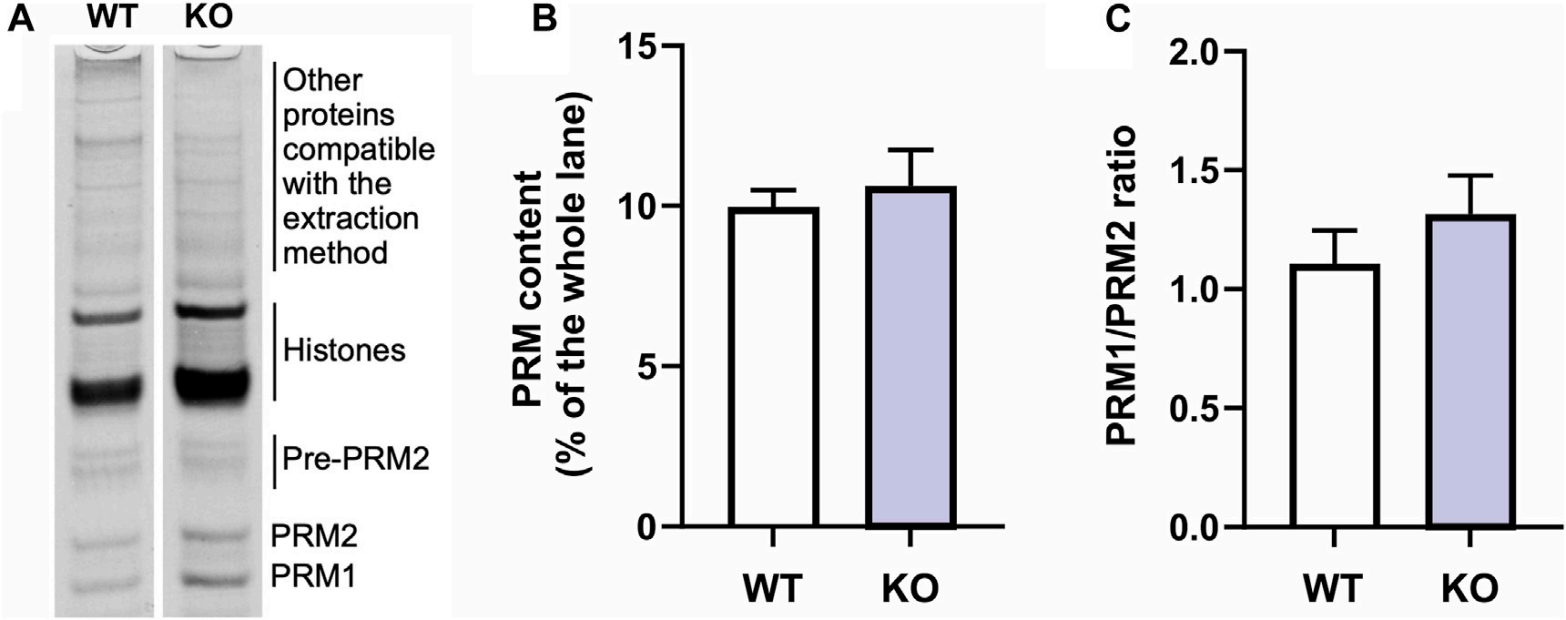
Comparison of protamine protein content between wild-type (WT) and *Spag17* knockout (KO). **(A)** Representative lanes of the acid-urea polyacrylamide gel indicating the bands corresponding to protamines (PRM1 and PRM2; pre-PRM2 = PRM2 precursor), histones and other extracted proteins. **(B)** Quantification showing protamine content as measured by protamine band density on a Coomassie blue stained acid-urea polyacrylamide gel as percent of the whole lane (*p* = 0.64). **(C)** Quantification of PRM1/PRM2 ratio (*p* = 0.38). Results are means ± SEM, n = 3 per genotype.

In order to explore the presence of defects at the protein expression level, we conducted an electrophoresis analysis of total proteins extracted from mouse testes, followed by relative quantification of protamine bands. The results indicated no significant difference in protamine content or PRM1/PRM2 ratio between the wild-type and knockout samples (Figure 4, Supplementary Figure S6). These findings suggest that the observed protamination defects are likely attributed to mechanisms other than protein expression levels.

### 3.3 SPAG17 is important for nuclear translocation of protamines

**FIGURE 5:**
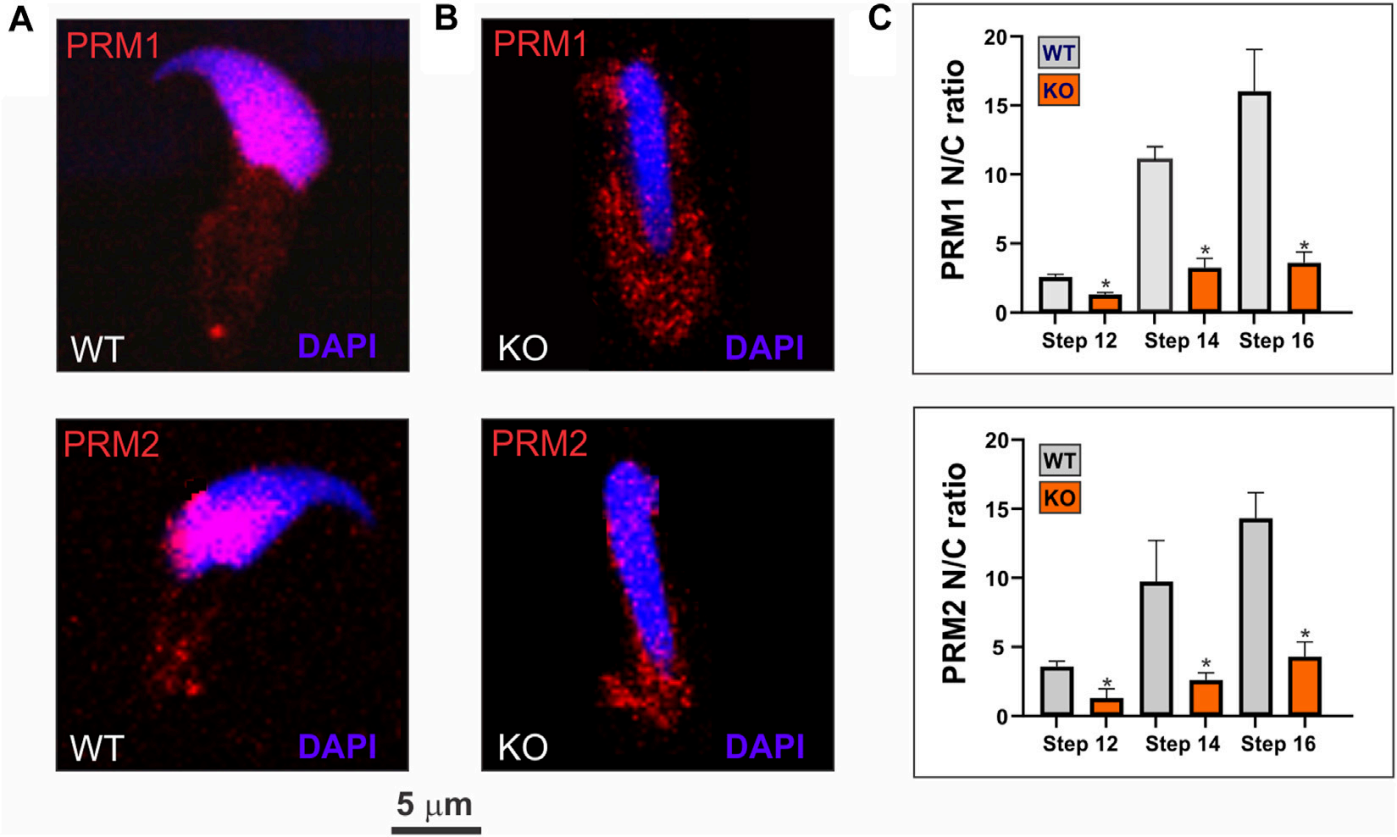
SPAG17 is important for nuclear translocation of protamines in spermatids. Spermatids were isolated from mouse testes and immunolabeled using anti-PRM1 and PRM2 antibodies. **(A)** Representative immunolabeling in wild-type (WT) spermatids showing nuclear localization of PRM1 and PRM2. **(B)** Representative immunolabeling in *Spag17* knockout (KO) spermatids revealed disrupted nuclear translocation of PRM1 and PRM2 **(C)** Quantification of nuclear/cytoplasmic (N/C) ratio of PRM1 and PRM2 localization in WT (n = 4) and KO (n = 4) spermatids at different steps during spermiogenesis. Results are means ± SEM. *Significant differences in comparison to WT; *p* < 0.05.

To understand the association of SPAG17 with PRM1 and PRM2, we investigated whether SPAG17 is necessary for the transport of protamines. Thus, nuclear translocation of PRM1 and PRM2 was studied by immunofluorescence using anti-PRM1 and PRM2 antibodies in wild-type and *Spag17* knockout spermatids. Figures 5A,B show PRM1 and PRM2 nuclear localization in wild-type spermatids. In contrast, these proteins were mainly detected in the cytoplasm of *Spag17* knockout spermatids, indicating reduced nuclear translocation. Quantification of the nucleus/cytoplasm ratio of PRM1 and PRM2 in spermatids from steps 12 to 16 showed reduced ratio in *Spag17* knockout spermatids in comparison to wild-type (Figure 5C).

**FIGURE 6:**
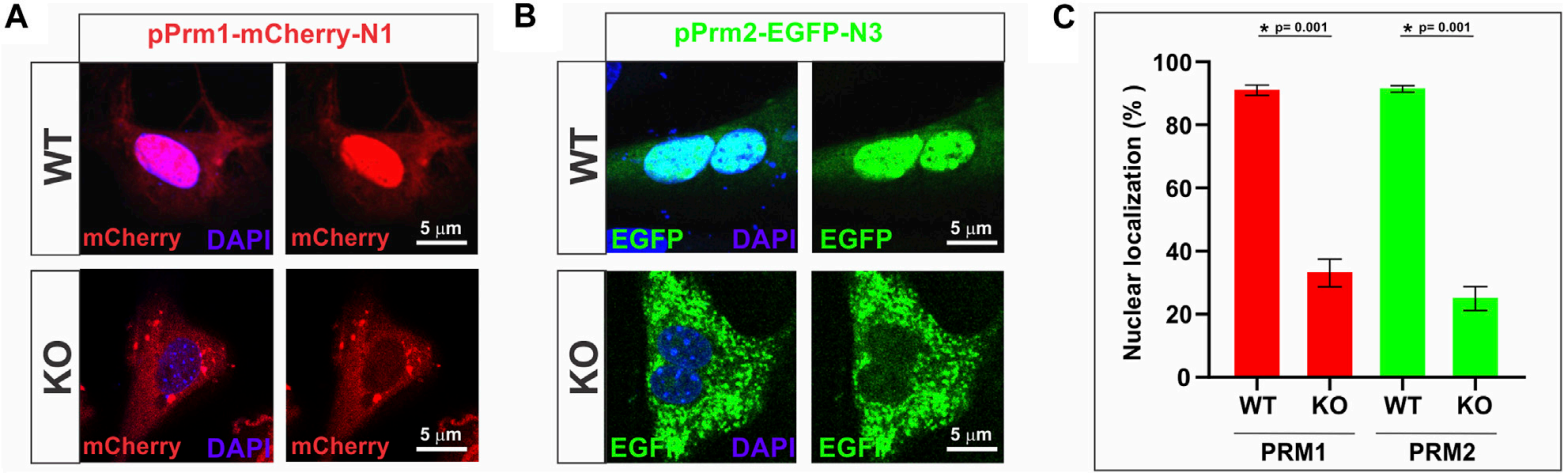
Transport of protamines into the nucleus is disrupted in the absence of SPAG17 in cultured fibroblasts. Mouse embryonic fibroblasts (MEFs) collected from wild-type (WT) and *Spag17* knockout (KO) embryos were transfected with mouse pPrm1-mCherry-N1 and mouse pPrm2-EGFP-N3 expressing vectors. **(A)** Representative images showing protamine localization in WT and KO MEFs after 24 h transfection with the PRM1 vector. **(B)** Representative images showing protamine localization in WT and KO MEFs after 24 h transfection with the PRM2 vector. **(C)** Quantification of nuclear localization of protamines. At 24 h post-transfection, the percentage of nuclear localization of PRM1 or PRM2 is significantly lower in *Spag17* knockout MEFs. Results are means ± SEM from four independent experiments. *Significant differences in comparison to WT; *p* = 0.001).

Building on this observation, we further explored the importance of SPAG17 in the transport of protamines using a fibroblast *in vitro* system. Mouse embryonic fibroblasts (MEFs) collected from wild-type and *Spag17* knockout embryos were transfected with mouse pPrm1-mCherry-N1 and mouse pPrm2-EGFP-N3 expressing vectors. pmCherry-N1 or pEGFP-N3 empty vectors were used as control vectors (Supplementary Figure S7). Figures 6A,B show that the localization of PRM1 and PRM2 is mostly in the nucleus of wild-type MEFs 24 h post-transfection. However, this does not occur in *Spag17* knockout MEFs, where protamines predominantly remain in the cytoplasm. Quantification of nuclear localization of PRM1 and PRM2 showed a significant difference (*p* = 0.001, for both PRM1 and PRM2, n = 4) between wild-type and *Spag17* knockout MEFs (Figure 6C). These results indicate that SPAG17 is required for transport of protamines from the cytoplasm to the nucleus. Due to the crucial role of protamines in DNA condensation, we conducted experiments to explore the subcellular localization of protamines within the nucleus. Interestingly, in our initial experiments, we did not observe noticeable areas enriched in protamines as markers for DNA condensation, as previously shown (Arévalo et al., 2022). To investigate further, we examined the time-dependent effect of protamines’ subcellular localization after 48 h post-transfection. Remarkably, we observed a significant enrichment of protamines in distinct areas within the nucleus (Supplementary Figure S8), confirming that this phenomenon is indeed time-dependent in wild-type MEFs. Conversely, after 48 h, protamines remain predominantly localized in the cytoplasm in *Spag17* knockout MEFs.

## 4 Discussion

During spermiogenesis, spermatids undergo a dramatic transformation from a round morphology to an asymmetric and elongated shape which is accompanied by DNA compaction and nuclear remodeling. In order to undergo such “metamorphic” transformations, spermatids have a complex but well-articulated system that delivers proteins to specific subcellular domains. In this context, a transitory structure named the manchette is assembled and serves as a track for protein trafficking (Kierszenbaum, 2002). Several proteins have been shown to localize to the manchette (Pleuger et al., 2020; Teves et al., 2020) but the function of a good number of these proteins and their interactomes are mostly unknown. We have previously shown that SPAG17, a protein originally characterized as a central pair protein in the flagellar axoneme (Zhang et al., 2005), is also associated with the manchette and is important for protein trafficking (Kazarian et al., 2018). Because earlier observations by transmission electron microscopy showed disrupted chromatin condensation in *Spag17* knockout spermatids (Kazarian et al., 2018), we hypothesized that there could be interactions between SPAG17 and protamines and that SPAG17 may be important for protamine trafficking.

The results presented here revealed interactions between SPAG17 and PRM1 and PRM2 with PLA and IP/MS. Remarkably, these proteins seem to interact in the cytoplasm and the nuclear area of elongating spermatids at the time when protamines are translocated into the nucleus.

Protamination was assessed in spermatids at different stages and in mature sperm as a measure of chromatin compaction promoted by protamines. It was anticipated that less compaction would translate into a higher proportion of cells staining with chromomycin A3 (Sakkas et al., 1995; Lolis et al., 1996). Our results indicated that protamination deficiency is significantly higher in the absence of SPAG17 in spermatids and in mature spermatozoa, similar to other knockout mouse models associated with defective DNA compaction (Yassine et al., 2014), and consistent with the original observations of disrupted chromatin condensation in the *Spag17* knockout (Kazarian et al., 2018). Deficiency of protamination could be due to decreased levels of protamine synthesis, or it may relate to transport of these proteins into the nucleus. Our data showed that the decrease in protamination is not due to differences in available protamines based on the quantitation of mRNA expression levels. Lack of *Spag17* did not affect the expression of *Prm1* and *Prm2*, since no differences were observed in mRNA levels of both genes. In addition, the ratio of *Prm1/Prm2* mRNA was also not different. Moreover, no differences were detected at the protein level. These findings suggest that reduced protamines levels in the nucleus were not related to their expression patterns but, rather to altered translocation into the nucleus.

To further investigate the possibility of disruptions in the transport of PRM1 and PRM2 from the cytoplasm to the nucleus, the distribution of these proteins in spermatids at different stages of spermiogenesis was analyzed. In the absence of SPAG17, PRM1 and PRM2 distribution was more abundant in the cytoplasm of spermatids from steps 12 to 16 in comparison to wild-type mice. This suggests that SPAG17 may have an active role in the translocation of protamines from the cytoplasm to the nucleus.

Additional evidence for the influence of SPAG17 in protamine translocation was obtained from *in vitro* studies using fibroblasts transfected with PRM1 or PRM2. Previous studies have characterized this fibroblast model system to understand the mechanisms of nuclear remodeling and reprogramming (Iuso et al., 2015). In sheep or mouse fibroblasts, transfection with human or mouse PRM1 results in gene silencing, nuclear shape changes and chromatin compaction (Iuso et al., 2015; Czernik et al., 2016; Palazzese et al., 2018). These changes have biological relevance because they resembled reprogramming and nuclear reorganization taking place in spermatids during differentiation (Iuso et al., 2015; Czernik et al., 2016). Similar results were found with transfection of PRM2 into HEK293 cells, where PRM2 localized to the somatic cell nuclei and a few nuclei seemed to be fully condensed (Arevalo et al., 2022a).

Our results showed that mouse embryonic fibroblasts from wild-type mice transfected with either PRM1 or PRM2 exhibited a very high percentage of cells with protein localized to the nuclei, in agreement with earlier results (Iuso et al., 2015; Arévalo et al., 2022a). However, in fibroblasts from SPAG17 deficient mice, there was a diminished proportion of nuclei with protamine, with the majority of PRM1 or PRM2 remaining in the cytoplasm even after 48 h post transfection. This provides additional evidence for the protamine transport dependency of SPAG17. Other proteins have been shown to be important for protein trafficking during spermiogenesis (Pleuger et al., 2020). Knockout models for some of these proteins lead to deformities in the nucleus (Teves and Roldan, 2022). However, it is unknown whether there is also altered chromatin condensation or if there are any interactions between these proteins and protamines.

Classical transport of biomolecules to the nucleus is mediated via the nuclear pore complex (NPC). This process involves several proteins including importins, nucleoporins, and a gradient of the small GTPase Ran between the nucleus and the cytoplasm (Miyamoto et al., 2012). Importins are a group of proteins with the capacity to bind a cargo and translocate it through nuclear pores. Selective cargo transport is possible because individual importins preferentially bind specific cargoes (Nathaniel et al., 2022). Importantly, several importins have been shown to play a role during spermatogenesis (Arjomand et al., 2014; Miyamoto et al., 2020; Liu et al., 2021; Nathaniel et al., 2022). Two proteins, RanGTPase-activating protein 1 (RanGAP1), in the cytoplasm, and RCC1, in the nucleus, maintain a gradient between the nucleus and the cytoplasm in mammals (Teves et al., 2020), which facilitate the nuclear internalization of the importins and the cargo protein through the NPC. Remarkably, RanGap associates to the manchette before transporting the protein complex from the cytoplasm to the nucleus (Kierszenbaum et al., 2002). Then, high nuclear RanGTP levels dissociate the importin-cargo complex, and the cargo is thereby positioned to effect nuclear roles (Nathaniel et al., 2022). To date, it is unknown whether any of these proteins interact with PRM1 or PRM2.

In conclusion, we showed that the transport of protamines is dependent on SPAG17. The identification of other proteins that are members of the interactome involved in this transport process requires further investigation. It is not known if SPAG17 is involved in the transport of other proteins into the nucleus including histones or transition nuclear proteins. The incomplete replacement of histones and/or aberrant PRM1 to PRM2 ratios, which are associated with sperm nuclear abnormalities, along with increased DNA fragmentation and decreased male fertility (Teves and Roldan, 2022), could arise from intricate interactions involved in the translocation of these proteins. Hence, understanding the mechanisms governing the nucleocytoplasmic transport of protamines holds significant importance for gaining a deeper insight into the underlying causes of male gamete dysfunction and infertility.

## Data Availability

The raw data supporting the conclusions of this article will be made available by the authors, without undue reservation.
