# Supplementary material for: SPAG17 mediates nuclear translocation of protamines during spermiogenesis
Source: Front Cell Dev Biol. 2023 Sep 12;11:1125096. doi: 10.3389/fcell.2023.1125096 (PMC10520709; doi:10.3389/fcell.2023.1125096)
Supplement: Supplementary file 1 [file DataSheet1.pdf]

SPAG17 mediates nuclear translocation of protamines during spermiogenesis

Clara Agudo-Rios[1,2†], Amber Rogers[1†], Isaiah King[1], Virali Bhagat[1], Le My Tu Nguyen[1], Carlos Córdova-Fletes[3], Diego Krapf[4], Jerome F Strauss III[5], Lena Arévalo[6], Gina Esther Merges[6], Hubert Schorle[6], Eduardo R S Roldan[2], Maria Eugenia Teves[1*]

[1] Department of Obstetrics and Gynecology, Virginia Commonwealth University, Richmond, USA

[2] Department of Biodiversity and Evolutionary Biology, Museo Nacional de Ciencias Naturales, CSIC, Madrid, Spain.

[3] Departamento de Bioquímica y Medicina Molecular, Facultad de Medicina, Universidad Autónoma de Nuevo León, Monterrey, Mexico.

[4] Department of Electrical and Computer Engineering, Colorado State University, Fort Collins, USA.

[5] Department of Obstetrics and Gynecology, Perelman School of Medicine, University of Pennsylvania, Philadelphia, USA.

[6] Department of Developmental Pathology, Institute of Pathology, University Hospital Bonn, Bonn, Germany.

Supplementary Material

Supplementary Figure S1. Schematic representation of PLA.

Supplementary Figure S2. Coomassie blue stained gel.

Supplementary Figure S3. Cell viability assessed using trypan blue staining.

Supplementary Figure S4. Map of the expression vectors used in this study.

Supplementary Figure S5. Proximity ligation assay (PLA) showing interaction of SPAG17 and protamines in mouse elongating spermatids.

Supplementary Figure S6. Coomassie blue stained acid-urea polyacrylamide gel used for the analysis of protamine content in wild-type (WT) and *Spag17* knockout (KO) testes.

Supplementary Figure S7. Mouse embryonic fibroblasts (MEFs) derived from wild-type (WT) embryos were subjected to transfection with mouse pmCherry-N1 and mouse pEGFP-N3 empty vectors.

Supplementary Figure S8. Time-dependent enrichment of protamines in distinct areas within the nucleus.

Supplementary Table 1. List of antibodies used for these studies.

Supplementary Video 1. Sperm motility in wild-type mice.

Supplementary Video 2. Sperm motility in *Spag17* knockout mice.

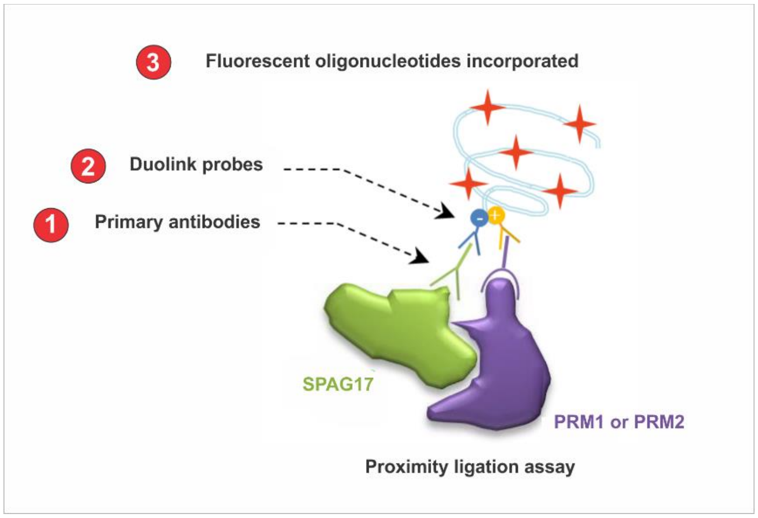

Supplementary Figure S1. Schematic representation of PLA. This technology, also referred to as Duolink® PLA technology, permits detection of protein-protein interactions in situ (at distances <40 nm) at endogenous protein levels. It exploits specific antibodies identifying (either directly or indirectly) the two proteins of interest and takes advantage of specific DNA primers covalently linked to the antibodies. A hybridization step followed by a PCR amplification with fluorescent probes then allows visualization of spots of proximity by fluorescence microscopy.

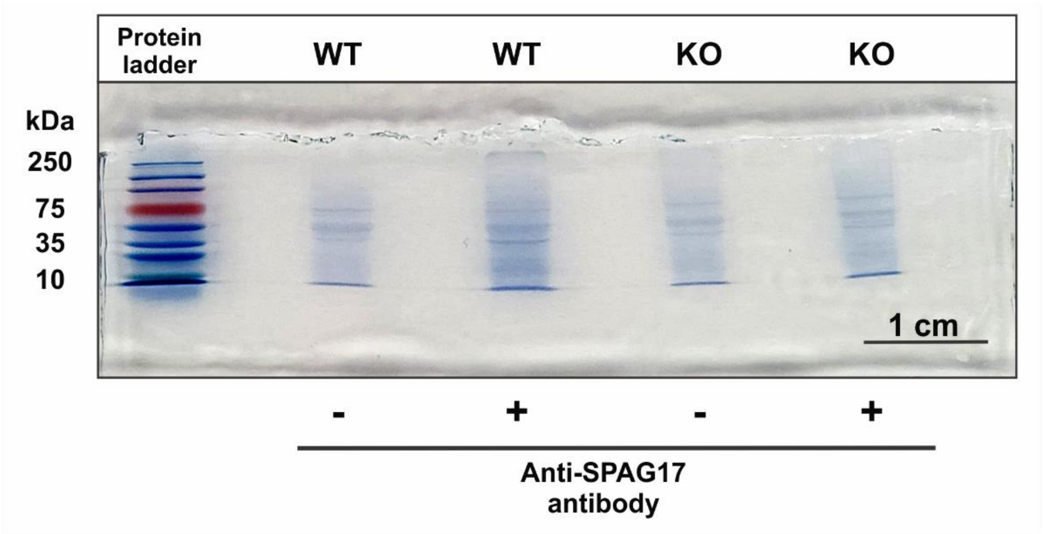

Supplementary Figure S2. Coomassie blue stained gel. After IP, proteins were loaded into a 10 % acrylamide gel well and electrophoretically separated. Representative gel from an IP experiment.

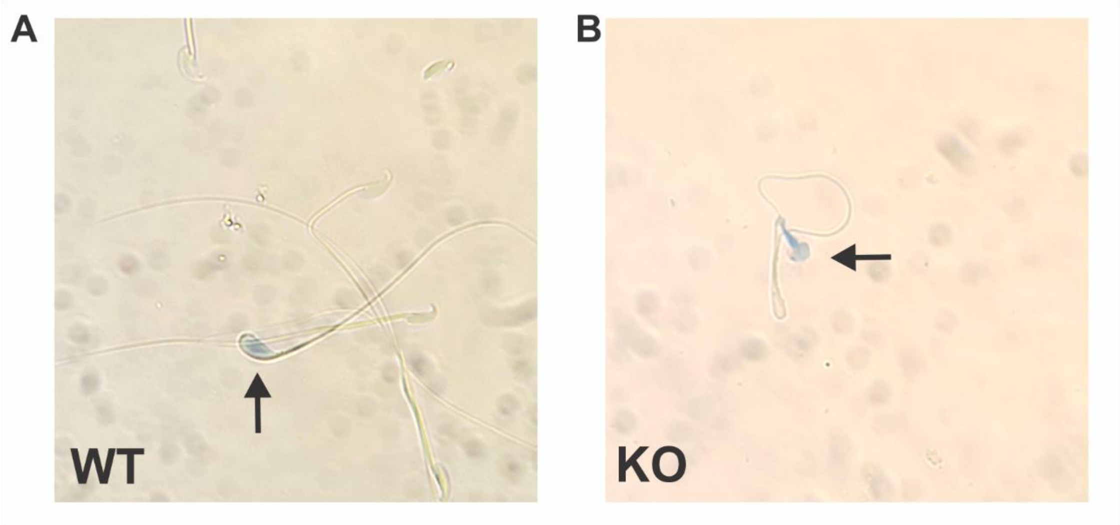

Supplementary Figure S3. Cell viability assessed using trypan blue staining. Viable cells maintain an intact plasma membrane, which excludes the trypan blue dye, while non-viable cells with compromised membranes allow the dye to cross (black arrow). An aliquot of the sperm suspension was stained with trypan blue, and the percentage of cells stained in blue was calculated to determine the percentage of dead cells. Representative images of both (**A**) wild-type (WT) and (**B**) *Spag17* knockout (KO) sperm stained with trypan blue captured by light microscopy.

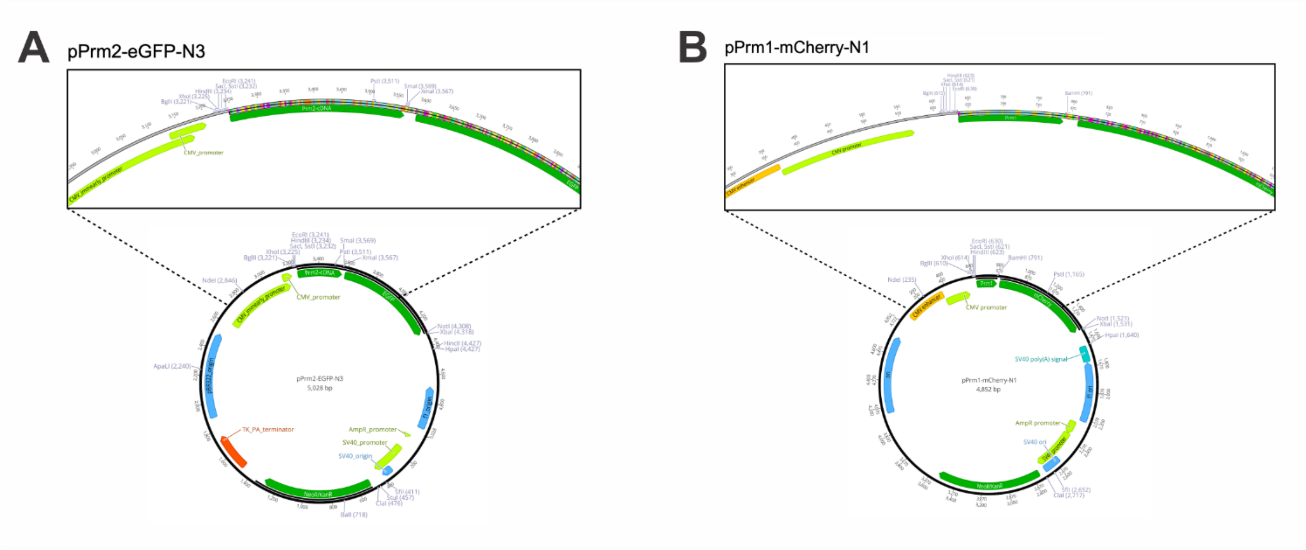

Supplementary Figure S4. Map of the expression vectors used in this study. (**A**) pPrm2-EGFP-N3. (**B**) pPrm1-mCherry-N1.

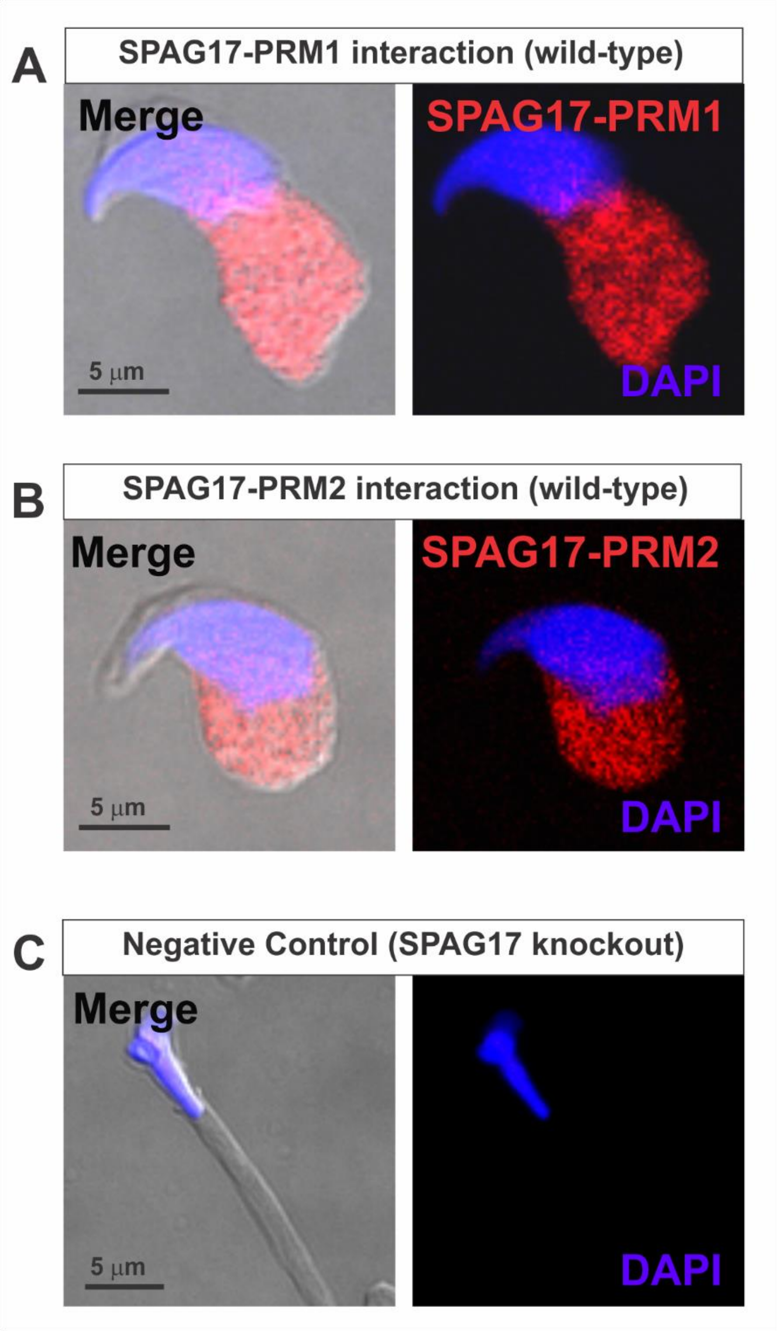

Supplementary Figure S5. Proximity ligation assay (PLA) showing interaction of SPAG17 and protamines in mouse elongating spermatids. (**A**) Representative image showing interaction of SPAG17 and PRM1 in wild-type elongating spermatid step 11-12. (**B**) Representative images showing interaction of SPAG17 and PRM2 in wild-type elongating spermatid step 11-12. (**C**) Representative images showing lack of interaction in *Spag17* knockout elongating spermatid step 14 when anti-SPAG17- and anti-PRM1antibodies were used in the absence of SPAG17 protein. Images were collected from 3 independent PLAs experiments.

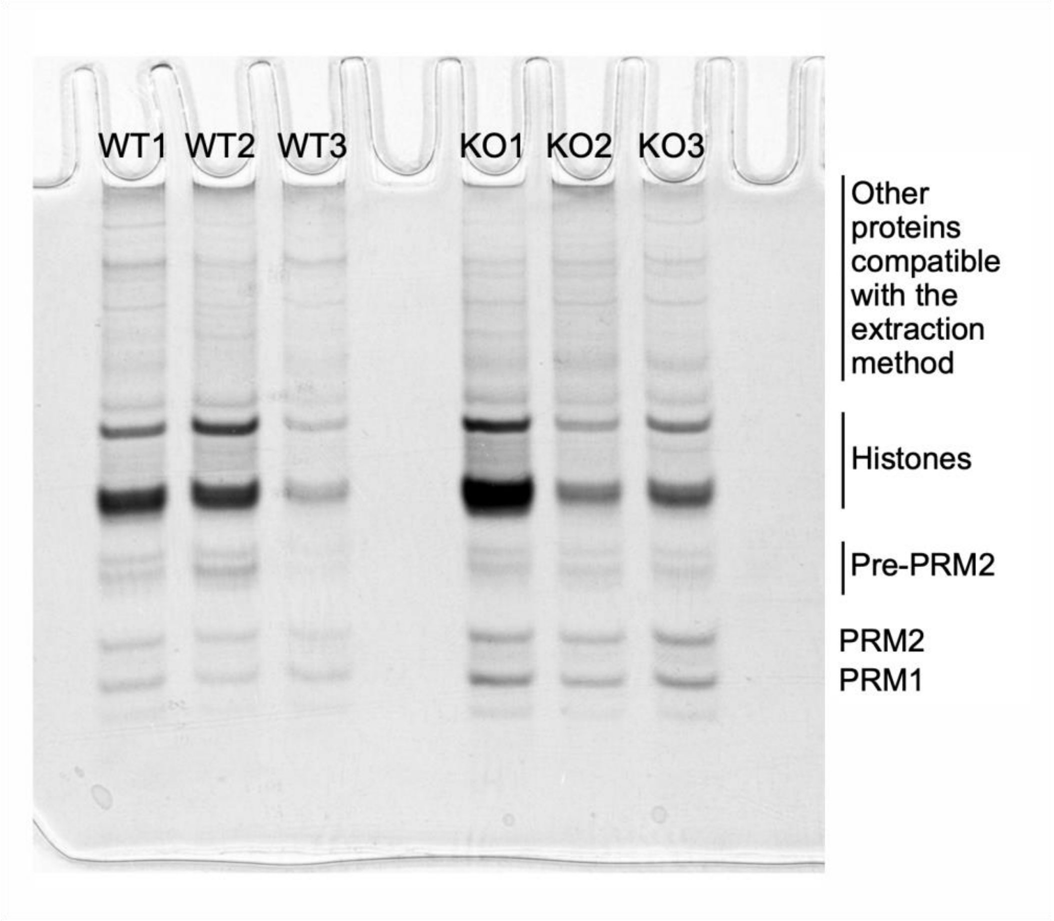

Supplementary Figure S6. Coomassie blue stained acid-urea polyacrylamide gel used for the analysis of protamine content in wild-type (WT) and *Spag17* knockout (KO) testes.

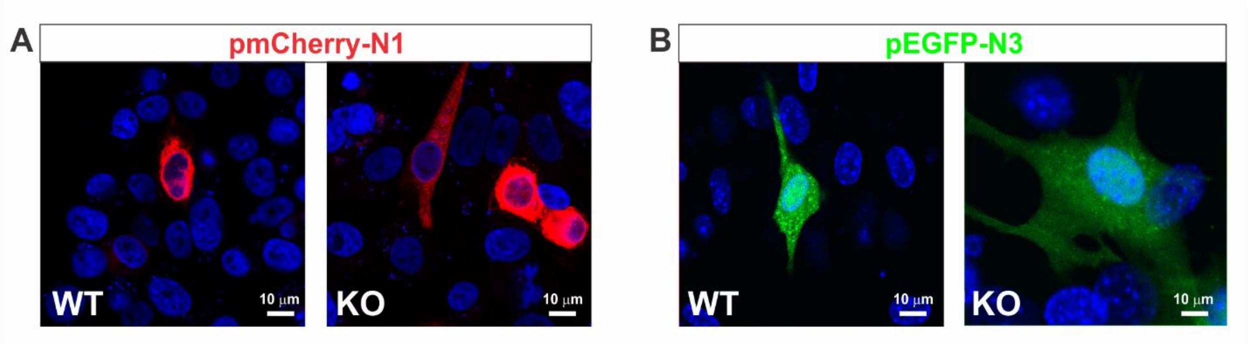

Supplementary Figure S7. Mouse embryonic fibroblasts (MEFs) derived from wild-type (WT) embryos were subjected to transfection with mouse pmCherry-N1 and mouse pEGFP-N3 empty vectors. After 24 hours of transfection, representative images were captured, showcasing the expression of pmCherry-N1 (**A**) and pEGFP-N3 (**B**) in the transfected MEFs; n=3.

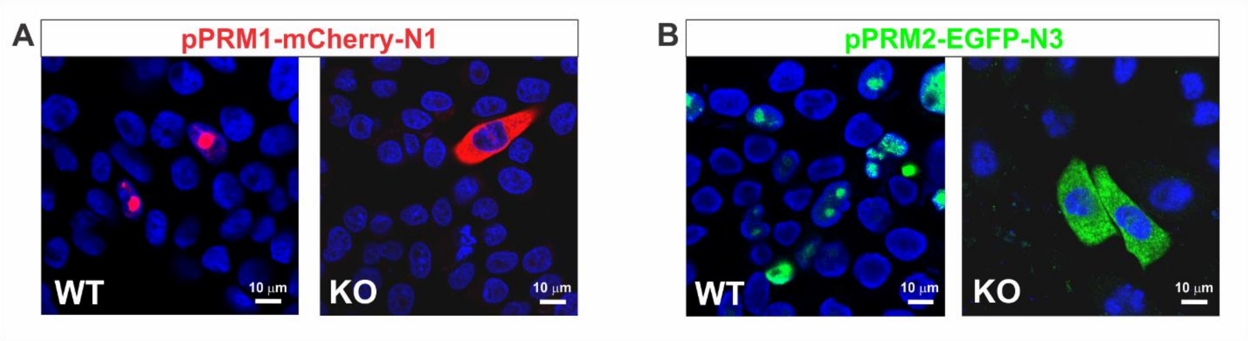

Supplementary Figure S8. Time-dependent enrichment of protamines in distinct areas within the nucleus. Enrichment of protamines in distinct areas within the nucleus was investigated in mouse embryonic fibroblasts (MEFs) obtained from wild-type (WT) and *Spag17* knockout (KO) embryos. The MEFs were transfected with mouse pPrm1-mCherry-N1 and mouse pPrm2-EGFP-N3 expressing vectors. After 48 h of transfection, representative images were captured, illustrating protamine localization in WT and KO MEFs for both the PRM1 vector (**A**) and the PRM2 vector (**B**); n=3.

Supplementary Table 1. List of antibodies used for these studies.

Antibody	Source	Specie	Dilution
Anti-SPAG17	Zhang et al., 2005	Rabbit	1/200
Anti-PRM1	Briar Patch Bioscience (Hup1N)	Mouse	1/100
Anti-PRM2	Briar Patch Bioscience (Hup2B)	Mouse	1/100
Anti-αTubulin	ThermoFisher Scientific (#A11126)	Mouse	1/200
Anti-βTubulin	Invitrogen (#PIPA521826)	Rabbit	1/200
Alexa Fluor 594-conjugated AffiniPure Fab Fragment Donkey Anti-Mouse IgG (H+L) antibody	Jackson ImmunoResearch Laboratories (715-587-003)	Donkey	1/3000

Supplementary Video 1. Sperm motility in wild-type mice. Sperm from adult wild-type (n=3) mice were collected from the caudae epididymides in pre-warmed (35 °C) Medium 199 supplemented with 4 mg/ml BSA and observed using phase contrast light microscopy.

Supplementary Video 2. Sperm motility in *Spag17* knockout mice. Sperm from adult *Spag17/Sox2-Cre* knockout (n=4) mice were collected from the caudae epididymides in pre-warmed (35 °C) Medium 199 supplemented with 4 mg/ml BSA and observed using phase contrast light microscopy.
